# Effects of a Preseason Neuromuscular Training Program vs. an Endurance-Dominated Program on Physical Fitness and Injury Prevention in Female Soccer Players

**DOI:** 10.1186/s40798-024-00731-7

**Published:** 2024-06-26

**Authors:** Ali Belamjahad, Claire Tourny, Nidhal Jebabli, Cain C. T. Clark, Ismail Laher, Anthony C. Hackney, Urs Granacher, Hassane Zouhal

**Affiliations:** 1 CETAPS UR 3832 (Research Center of Sport Science), University of Rouen Normandy, Rouen, France; 2 High Institute of Sport and Physical Education of Kef, UR22JS01, University of Jendouba, Kef, Tunisia; 3 Institute for Health and Wellbeing, Coventry University, Coventry, CV1 5FB UK; 4 College of Life Sciences, Birmingham City University, B15 3TN Birmingham, UK; 5 Department of Anesthesiology, Pharmacology and Therapeutics, The University of British Columbia, Vancouver, Canada; 6 Department of Exercise & Sport Science, University of North Carolina, Chapel Hill, NC USA; 7 Department of Sport and Sport Science, Exercise and Human Movement Science, University of Freiburg, Freiburg, Germany; 8 Université Rennes, M2S (Laboratoire Mouvement, Sport, Rennes, Santé France; 9 Institut International des Sciences du Sport (2I2S), 35850 Irodouer, France

**Keywords:** Football, Exercise training, Strength, Women Soccer, Season

## Abstract

**Background:**

The pre-season preparatory period is considered key for optimizing the physical fitness levels needed to withstand congested match periods and preventing injuries during the regular soccer season. This study contrasted the effects s of neuromuscular training (NMT) versus an endurance-dominated training (ET) program conducted during the preseason on measures of physical fitness and injury occurrence in female soccer players.

**Methods:**

Twenty-four female soccer players aged 17.0 ± 1.3 years from a professional soccer club participated in this study. Players were randomly assigned to NMT (*n* = 12) or ET (*n* = 12) groups according to their playing position. The preseason intervention program lasted six weeks, with three weekly sessions with a duration of 45–60 min per session. Exercises in the NMT group included muscle strengthening exercises, plyometrics, agility and dynamic stability exercises, while the ET group practiced a traditional pre-season training program consisting of running and sprinting exercises, fartlek, and high-intensity interval training. The training volumes were similar in the two study groups. Anthropometric measurements, physical fitness tests (i.e., linear and change-of-direction speed, muscle strength and power tests) and the overall injury rate per 1000 h of exposure (training, match) were recorded throughout the season.

**Results:**

No between group differences were found at pre (T1). Significant group-by-time interactions were observed for the 5, 10, and 30-m linear sprint speed tests (*p* < 0.001, 2.16 < d < 2.58), the T-test (*p* = 0.024, d = 1.03), the squat (*p* < 0.001, d = 4.04), and the countermovement jump test (*p* < 0.001, d = 2.21), the Loughborough soccer passing test (LSPT) (*p* = 0.019, d = 1.08), and the 1-RM back squat test (*p* < 0.001, d = 2.53). Post-hoc tests indicated that NMT provided larger improvements for SJ, CMJ, 1-RM back squat, the 5-m sprint, 10-m sprint, 30-m sprint, T-test and LSPT compared to ET (1.07 > d > 2.77). The injury rate across the season was significantly lower in the NMT (5.1/1000 h exposure) compared to ET (11.8/1000 h exposure) (*p* = 0.014).

**Conclusions:**

The findings support that six-weeks of preseason NMT versus ET induced larger performance improvements, and significantly reduced injury occurrence in elite female soccer players.

## Key points

• Our results indicate that the integration of 45 to 60 min of preseason neuromuscular training, three times per week, for 6 weeks, can improve physical fitness and prevent injuries in young female soccer players.
• The present study demonstrated that the neuromuscular training program can reduce the incidence injury rate by more than 50% and the burden by more than 65% compared to the endurance-dominated training.
• Neuromuscular training is an effective approach during the preseason period to improve diverse measures of physical fitness and to reduce injury occurrence.
• Preseason neuromuscular training appears to improve the fitness level of female soccer players and as such could allow them to compete at the high level.

## Background

Football, or soccer, is widely recognized as an intermittent activity that requires players to undertake repeated high-intensity efforts over the match duration of 90–120 min [1]. Besides aerobic endurance, match play requires linear sprint and change-of-direction (CoD) speed (e.g., ball dribbling, accelerations, decelerations), muscle power related actions such as vertical jumping and landing [1], and tackles [2]. These physical fitness qualities and motor skills represent important performance determinants in soccer [1, 3].

The physical effort of soccer players can be measured through external (e.g., distance covered) and internal load metrics (e.g., heart rate, biological markers, rate of perceived exertion) during training or matches [1]. Evidence supports that over the past years, physical demands have increased in women’s soccer [4]. A study by Bradly and Scott [4], working in conjunction with FIFA, compared the physical demands of the 552 female soccer players during the World Cups in Canada (2015) and France (2019). While the average total running distance during a match was similar in both events, significantly more (16–32%) high-intensity runs (> 19 km/h) were noted in 2019 compared to 2015 with fewer (5%) low-intensity runs (< 13 km/h) in 2015 [4]. This trend for changes in the women’s game indicates that the preseason preparation period becomes even more important to develop adequate physical fitness levels to realize high training and match intensities during the soccer season and tournaments [4–8].

Preseason training is designed to develop players’ physical fitness and prepare them for the various demands during the competitive season [6, 7, 9]. Ekstrand et al. [9] demonstrated that athletes who completed more training volume during the preseason were less likely to sustain injuries during the in-season period. That is, preseason related improvements of physical fitness appear to protect players from the demands of the competitive soccer season [5, 9]. Hence, the aforementioned studies support the possible role for high-quality pre-season soccer training not only for developing physical fitness but also for injury prevention throughout the entire competitive period [10].

Traditionally, many soccer coaches adopt a pre-season endurance-dominated (ET) training program which includes a high proportion of low-intensity continuous endurance exercises including running at a constant or varied pace, as well as moderate- and high-intensity interval training. During the pre-season, friendly matches can also be classified as ET, technically similar to interval training in terms of physiological load, incorporated with soccer-specific technical and tactical skills [7]. The preseason period also foresees neuromuscular training (NMT) which is generally integrated in the late phase of the preparation period, using plyometrics and exercises with the goal to improve power, linear sprint and CoD speed [7]. ET has the potential to improve aerobic capacities (e.g., VO2max) of young soccer players [11] and may prevent injuries during the competitive period [12]. However, the application of ET can have a negative impact on the training-induced development of other soccer-related physical fitness qualities such as linear sprint or CoD speed [11]. Moreover, performing highly intensive interval training in-season [13] has shown a significant increase in aerobic capacity accompanied by a significant increase in 40-m sprint capacity.

Previous studies have reported that female soccer players show significantly lower physical fitness levels compared with their male counterparts [3]. That said, previous studies have also reported that NMT is well-suited to improve performance and reduce injuries in female soccer players [3, 14, 15], such as the FIFA 11 + training program [16]. Notably, NMT is a multimodal exercise regime that targets core stability, static and dynamic balance, weight transfer, coordination, rapid CoD, and muscle strength and power activities. Recently, Granacher et al. [17], postulated that the integration of NMT, either during the warm-up or during sport-specific training, has the potential to improve measures of muscle strength and power, linear sprint and CoD speed, agility, balance, cardiorespiratory fitness, and sport-specific performance in athletic and non-athletic populations [17]. This finding is supported by the published work of Roso-Moliner et al. [15], in elite female soccer players. Thus, it would be very interesting to compare the effects of NMT during the preseason to the traditional ET program [15].

To our knowledge, there is no study available that has examined the impact of NMT applied during the preseason on measures of physical fitness and injury occurrence in female soccer players. Accordingly, the aim of this study was to contrast the effects of NMT versus a traditional ET preseason program on selected measures of physical fitness and injuries in young female professional soccer players across a full soccer season. We hypothesized that NMT would be more effective than ET to improve physical fitness and to lower injury rates in female soccer players based on the findings of previous reports [10, 15, 18–20].

## Methods

### Participants

**Fig. 1:**
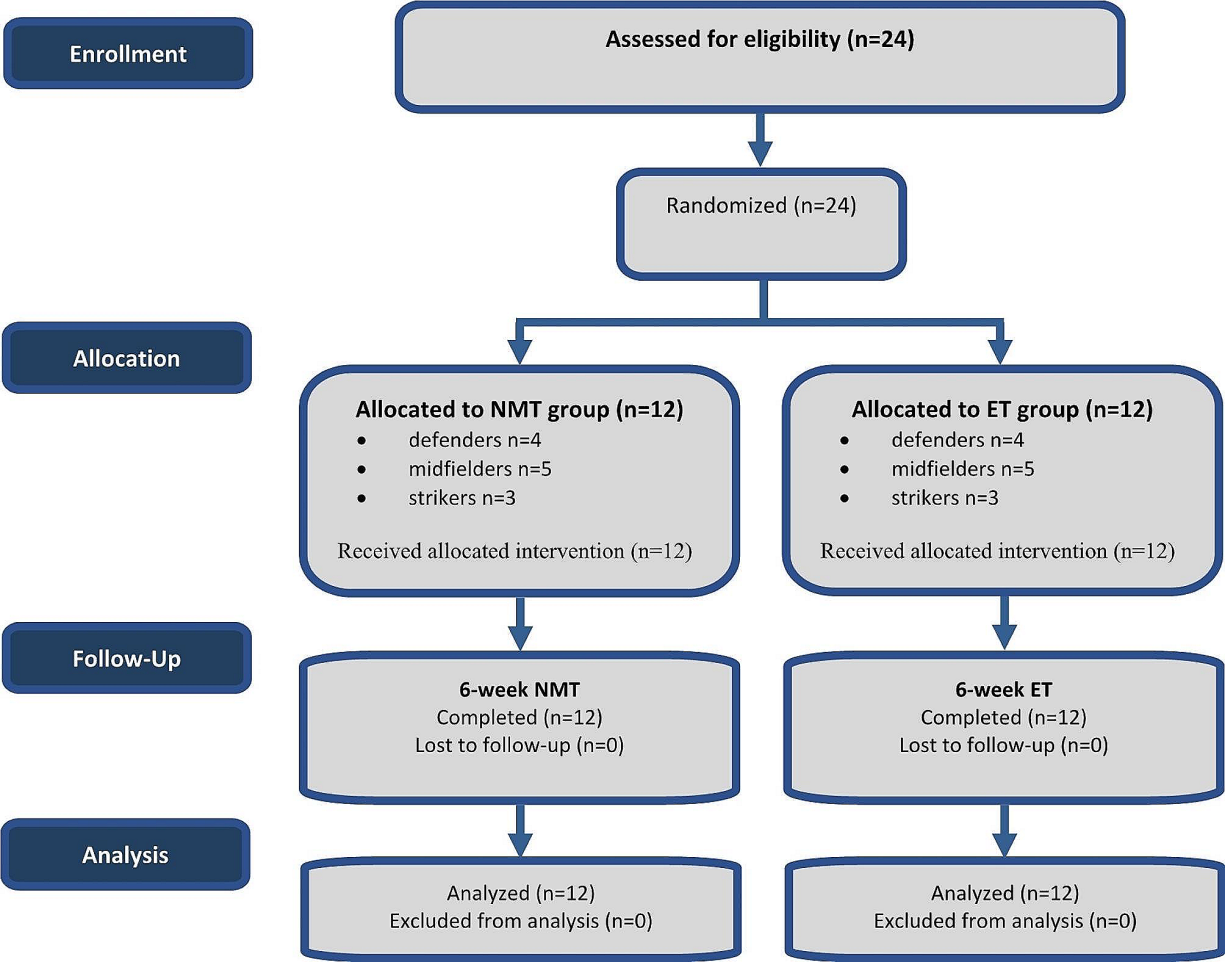
CONSORT flow diagram of participant recruitment. ET, Endurance-dominated training; NMT, Neuromuscular training

Twenty-four highly trained [21] female soccer players aged 16 to 18 years from a professional soccer club (Renaissance sportive de Berkane 1st Division, Berkane, Morocco) participated in this study. The players (8 defenders, 10 midfielders, and 6 strikers) were randomly allocated to the two experimental groups according to their playing positions ***(***Fig. 1***)***.

Players and their parents were informed about the study procedures, potential benefits, and risks before signing the consent form. The study was conducted in accordance with the latest version of the Declaration of Helsinki and local ethical approval was received from the Ethics Committee for Biomedical Research of Oujda (CERBO; ethical approval code: 05/2022), Morocco.

A minimal sample size was calculated using an a priori statistical power tool (G∗Power, Version 3.1, University of Dusseldorf, Germany). The a priori power analysis was computed for our primary outcome (i.e., 10-m linear sprint performance) with the following input parameters: 1-β of 0.90 (power), alpha level of 0.01, and large effect size (Cohen’s f = 0.499; Cohen’s d = 0.980; [2]). The results of this analysis indicated that a total sample size of *N* = 20 players would be required to achieve significant group-by-time interactions, given the above specified conditions. The sample size was increased by 20%, to 24, to allow for potential drop outs of study participants.

### Anthropometric Measurements

Participants performed anthropometric and physiological testing as well as physical fitness assessments before and after the 6-week preseason intervention program. Body height was measured using a stadiometer with an accuracy of 1 mm (SECA 206 ®), and body mass with an electronic scale with an accuracy of 0.1 kg (KINLEE ®). All players wore minimal clothing and no shoes during testing. Body fat percentage was measured using dual-energy X-ray absorptiometry (DEXA) using Lunar iDXA instrument (GE Medical Systems, Madison, Wisconsin) [22]. The same investigator completed all tests, adhering to the positions and procedures outlined by the International Biological Program. The physical characteristics data of the participants are presented in Table 1.

**Table 1:** Characteristics of the study participants

Group	Number	Age (years)	Body height (cm)	Body mass (kg)	Body fat (%)
ET	*n* = 12	17.0 ± 1.6	165.9 ± 5.4	60.2 ± 5.6	22.4 ± 3.0
NMT	*n* = 12	17.0 ± 1.3	164.3 ± 5.4	58.1 ± 4.5	21.3 ± 3.8

ET, endurance training group; NMT, neuromuscular training group; Data are presented as mean ± standard deviation

### Physical Fitness Tests

Field and laboratory-based tests were applied pre (T1) and post the six-week interventions (T2) to assess the impact of the two training methods on players’ physical fitness. Countermovement (CMJ) and squat jump (SJ) height were applied to assess proxies of muscle power using an optoelectric system (Optojump. Microgate. Bolzano. Italy). Maximal strength was tested with the help of a ten repetitions maximum test (10-RM). For the squat and bench press exercises, the 10-RM was used to estimate the 1-RM. Linear sprint speed (5-10-30-m), CoD speed tests (CoD with and without a ball), and soccer-specific qualities using a repeated shuttle sprint ability (RSSA) test, the Yo-Yo intermittent recovery test 1 (YYIRT 1), and the Loughborough soccer passing test (LSPT) (described below) were also included in the physical fitness test battery.

Physical fitness tests were scheduled during the week preceding the study. On the first day, the anthropometric measurements were obtained. On the second test day, the CoD (change of direction test) with and without ball and the YYIRT1 (Yoyo intermittent recovery test level 1) were assessed. On the third day, SJs and CMJs, linear sprints (5-10-30-m) were tested. On the fourth test day, the LSPT test and the RSSA (repeated-shuttle-sprint ability) were applied. The physical fitness tests were performed on a third-generation synthetic pitch and monitored by the same examiner. Pre and post tests were performed in the same test sequence and at the same time of day in sunny weather.

### Muscle Power and Muscle Strength

#### Muscle Power

##### Vertical Jump Tests

Proxies of lower limb muscle power were tested using SJs and CMJs. For the SJ, players remained in a static semi-squatted position with a 90° knee flexion angle for one second before executing a maximal vertical jump. For the CMJ, players started in an upright standing position and had to perform a downward movement to a semi-squatted position followed by a full and explosive vertical extension of the legs. Hands were held akimbo (hands on the hips and elbows turned outwards) during SJs and CMJs. Both CMJs and SJs were performed at maximal effort. Three test trials were performed for SJs and CMJs with a rest of 60 s between jumps. The average of the two best vertical jump heights was taken for further data analysis. For the assessment of jump height, flight time was used from the Optojump system (Opto Jump, Microgate, Italy) to deduce jump height. The players performed the jumps in the same test sequence (i.e., SJs followed by CMJs) during pre and post-tests [23]. The intra-class correlation coefficients (ICC) for test–retest reliability was 0.923 (0.822–0.967) for SJ and 0.961 (0.911–0.983) for CMJ.

##### Maximal Strength (1-RM)

Bench press and back squat 10-RM tests were used to estimate 1-RM and thus maximal strength. For the assessment of maximal strength, we estimated the 1-RM from the number of repetitions lifted (10-RM) at a given load to avoid the risk of injury at the start of the season. The 10-RM test protocol is similar to the 1-RM protocol, but each set requires ten repetitions. To estimate the players’ 1-RM from a submaximal load (10-RM = 75% of 1-RM), the prediction table proposed by Haff and Triplett was used [24]. The test started with a warm-up with a low load that easily allowed the completion of ten repetitions. The load was increased during each set of ten repetitions with a recovery time of 2–4 min between sets until the player could not complete the ten repetitions. The increase in load in each set did not exceed 5 to 10% of the upper body mass and 10 to 20% of the lower body mass. Ideally, the athlete’s 10-RM is measured in three to five repetitions. The estimation of the 1-RM from the 10-RM was based on methods as described by Sheppard and Triplett [25]. After the back-squat test, a 10-minute rest interval was allowed before the bench press test was started. No timing (tempo) was imposed for eccentric and concentric movement phases. The ICC for test–retest trial for RM and squat RM were 0.916 (0.806–0.964) and 0.875 (0.710–0.946), respectively.

##### Linear Sprint Speed

The players performed three maximal 30-m linear sprints, with split times of 5-m and 10-m recorded and a 1-min rest between each trial [26]. Players started each sprint 0.5 m before the first timing gate positioned at hip height of the participants. The split times at 5, 10, and 30-m were recorded using three photocell gates (Smartspeed, Warwickshire, United Kingdom) positioned at the prescribed distances from the first photocell gate. The fastest time of the three trials at 5, 10, and 30-m was recorded for the final analysis. The ICC for test–retest trial for 5-m, 10-m and 30-m performances was 0.825, 0.889 and 0.920, respectively.

##### Change-of-Direction Speed (CoD)

The “T-test” is a CoD test that assesses the capacity of players to quickly change directions. The test was performed according to the protocol described by Semenick [27]. The test includes various CoD movements starting with a forward sprint from point A to point B (9.14 m), a run to the left from point B to point C (4.57 m), a run to the right from point C to point D (9.14 m), a run to the left from point D to point B (4.57 m) and a backward run towards the starting point from point B to point A (9.14 m) with and without a ball. The four points (A, B, C, and D) form a large “T”. Players start the test with both feet behind the starting point A and complete the circuit as quickly as possible. The test time was recorded using the same photocell system as used during the linear sprint speed test. The players completed three trials and the fastest was used for further analysis. The ICC for test–retest trial for sprint with ball and sprint without ball, was 0.886 and 0.906 respectively.

### Soccer-specific Performance

#### Repeated Shuttle Sprint Ability (RSSA)

The RSSA assesses the potential to withstand the intermittent efforts of soccer and was adapted to the specific movements of soccer with rapid CoDs. After a standardized 15-minute warm-up session, the players performed 6 × 20–20 m (20 m outward and 20 m return) sprints at maximum speed interspersed with 20 s of passive recovery. Sprint time was recorded using the same photocell system as described in the previous tests. The best time was used for the analysis of the results (RSSA best). Thereafter, the percentage decrease in performance (RSSA decrement) was calculated according to the following formula: RSSA decrement = ([average RSSA] / [best RSSA] x 100) – 100) [28]. The ICC for test–retest reliability was 0.933 for RSSA best, and 0.907 for RSSA decrement.

#### The Yo-Yo Intermittent Recovery Test 1 (YYIRT 1)

This test assesses players’ endurance performance and their ability to repeat high-intensity efforts. The YYIRT 1 test was performed on an outdoor synthetic third generation pitch. The test consisted of two repeated 20-m runs (round trip) at progressively increasing speed controlled by an audible signal from a tape recorder and separated by a 10-second recovery time. The running speed increased in stages and the players continued the test until they were unable to keep up with the rhythm imposed by the audio file. When a player failed to reach the finish line in time on two consecutive occasions, the distance covered (m) was recorded as the test result. The test was preceded by a standardized 10 min warm-up [29]. The ICC for test–retest reliability was 0.988 for the distance covered.

#### Loughborough Soccer Passing Test (LSPT)

**Fig. 2:**
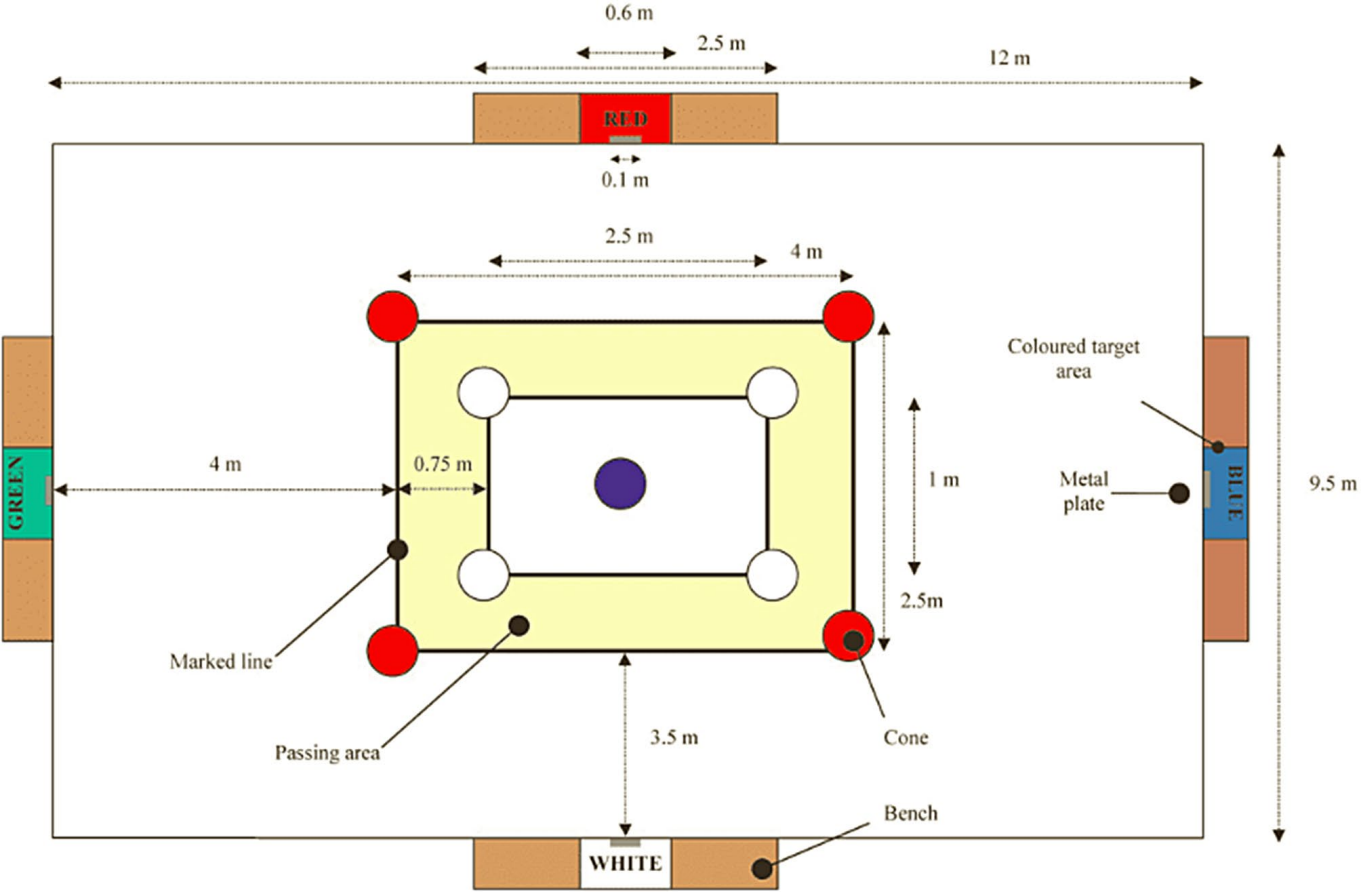
Diagrammatic representation of the Loughborough soccer passing test (LSPT) [30]

This test was developed and validated for research purposes by Ali and colleagues [30] to assess soccer specific technical performance (Fig. 2). The players started the LSPT with the ball at the center point of the test space, and then they were asked to complete 16 passes (about 4-m distance) against benches placed in a rectangle around the player. A colored piece of cardboard (60 × 30 cm) was attached to the center of each bench, serving as a target area (red, blue, green, and white), and passes were made in one of four random color orders selected by an interviewer. Players were asked to complete all 16 passes as quickly as possible and attempting to minimizing errors. The sequence of passes was determined by one of eight trial commands randomly generated by the investigators such that each trial consisted of eight long passes (4 m; green and blue) and eight short passes (3.5 m; white and red). LSPT performance includes the original time to complete all 16 passes, penalty time (time added for errors, inaccurate passes), and performance time (original time + penalty time). The penalty time was calculated using the following criteria: +5 s for completely missing the bench or passing to the wrong bench/ +3 s for missing the target area (60 × 30 cm)/ +3 s to ball handling/ +2 s to pass the ball from outside the passing zone/ +2 s if the ball hits a cone/ +1 s for each second taken out of the 43 s allocated to complete the test/ − 1 s for each pass that hits the 10 cm band in the middle of the target (bonus). Players were asked to complete the test as quickly as possible with the fewest errors to optimize performance in the LSPT, and were permitted two trials, the average of which was used as the performance score [30]. The ICC for test–retest reliability for total time (TT), P and T was 0.856, 0.935, and 0.949, respectively.

### Training Load Quantification

#### Internal Training load: Ratings of Perceived Exertion (RPE)

The internal training load was estimated using Borg’s session rating of perceived exertion (session-RPE) adapted from 0 to 10 for the 15–20-minute period after the end of each training session within the interventions. The players were familiar with the Borg scale from experiences during the previous season. Players had to complete the RPE within 15–20 min following the session. The RPE was calculated according to Foster et al. [31] by multiplying the intensity from the Borg scale by the duration of the session in arbitrary units (AU).

#### External Training Load: Global Positioning System (GPS)

The external load of the player can be estimated by quantifying the distances covered at various levels of intensities (walking, light running, moderate running, high intensity running, sprinting, acceleration, deceleration) [1], and was measured using a GPS system (Gpexe®. Udine, Italy) after each session and match during the intervention period. It was demonstrated that the Gpexe®. provide valid and reliable movement orientation data for team-sport locomotion [32]. Distances were quantified from speed data derived from the GPS system. The speed zones used by the Fédération Internationale de Football Association (FIFA) to analyze the 2015 and 2019 World Cups were adopted as follows: Zone 1 (0–7 km/h), Zone 2 (7–13 km/h), Zone 3 (13–19 km/h), Zone 4 (19–23 km/h), and Zone 5 (> 23 km/h). The recorded distances were reported as absolute: total distance, high-speed run (Zone 3), very high-speed run (Zone 4), sprint (Zone 5), and maximum speed. The number of sprints (> 23 km/h), acceleration (+ 3 m/s²), and deceleration (-3 m/s²) were assessed.

### Intervention Program

The players completed six to eight training sessions per week during the six-week preseason training period (i.e., intervention). The training sessions took place in the morning ∼ 9 am (∼ 45–60 min) and in the afternoon ∼ 5 pm (∼ 60–80 min). A minimum of 48 h rest was granted between the exercise sessions (NMT, ET). All training sessions were supervised by a professional fitness coach. The morning sessions were mainly reserved for the development of physical fitness. Training volumes were similar between the two intervention groups.

#### Group 1: Endurance-dominated Training (ET)

Twelve female soccer players followed a traditional preseason ET program *(*Table 2*)*. The sequence of physical training foresaw an exercise emphasis on aerobic capacity during the first two weeks. During weeks 1, 2 and 3, we focused on the development and maintenance of the aerobic capacity. The speed endurance was developed and maintained during weeks 4, 5 and 6.

**Table 2:** Endurance-dominated training program description

Session 1	Session 2	Session 3
**Endurance 45’** Running without a ball 3 × 10’ (70–80% HRmax) *R* = 2’ Stretching 10’	**Endurance 45’** Running with a ball 3 × 12’ (70–80% HRmax) *R* = 2’ Stretching 5’	**Endurance 60’** Running with a ball 3 × 15’ (70–80% HRmax) *R* = 2’30 Stretching 10’
**Endurance 60’** Warm-up/circuit drills 25’ 5 × 4’ interval training (75–85% HRmax) *R* = 2’ Stretching 5’	**Endurance 60’** Warm-up 15’ 5 × 4’ interval training (75–85% HRmax) *R* = 2’ Stretching/flexibility 15’	**Endurance 60’** Warm-up/circuit drills 20’ 3 × 8’ Fartlek (1’-1’) (75–85% HRmax) *R* = 4’ Stretching 5’
**Endurance 60’** Warm-up/ aerobic circuit 20’ 3 × 8’ Fartlek (1’-1’) (75–85% HRmax) *R* = 3’ Stretching 5’	**Endurance 60’** Warm-up/coordination 25’ 2 × 8’ aerobic circuit Interval training 30–30 s (90% MAS) *R* = 4’ Stretching 10’	**Endurance 60’** Warm-up/aerobic circuit 30’ 3 × 6’ Intermittent 15–15 s (95% MAS) *R* = 3’ Stretching 5’
**Endurance 60’** Warm-up/aerobic circuit 30’ 2 × 8’ Interval training 15–15 (100% MAS) *R* = 4’ Stretching 8’	**Speed endurance 60’** Warm-up/aerobic circuit 20’ Speed endurance 30’ Stretching 10’	**Endurance 60’** Warm-up/aerobic circuit 30’ 3 × 6’ Intermittent 15–15 (100% MAS) *R* = 3’ Stretching 5’
**Speed endurance 60’** Warm-up/aerobic circuit 20’ Speed endurance 30’ Stretching 10’	**Endurance 60’** Warm-up/aerobic circuit 20’ Interval training 2 × 6’ (10–20 s) / 1 × 6’ (10–10 s) (110% MAS) *R* = 3’ Stretching 15’	**Speed endurance 60’** Warm-up/aerobic circuit 20’ Speed endurance 30’ Stretching 10’
**Speed endurance 45’** Warm-up/aerobic circuit 20’ Speed endurance 30’ Stretching 10’	**Endurance 45’** Warm-up 10’ Interval training 1 × 6’ (10–20 s) / 1 × 6’ (5–25 s) (120% MAS) *R* = 3’ Stretching 15’	**Speed endurance 45’** Warm-up/aerobic circuit 20’ Speed endurance 30’ Stretching 10’

HRmax, maximum heart rate; R, recovery; s, seconds

#### Group 2: Neuromuscular Training (NMT)

The NMT group (*n* = 12) exercised for six weeks with three weekly sessions, including a warm-up (Table 3). Each session lasted 45 to 60 min. The volumes and intensities were gradually increased to avoid overtraining or injury to the players. The NMT program incorporated general strengthening, plyometrics, agility, speed, core stability, and balance (dynamics and lumbo-pelvic control) exercises. The fitness coach kindly advised players to perform the exercises with correct movement technique (e.g., lower limbs alignment).

**Table 3:** Neuromuscular training program description

	Distance	Repetition/Time					
**Warm-up (7’ and 10’)**							
- Joint mobility (Spine, hip, knee, ankle)		2 × 15 s per joint					
- High knees, reps	10 m	2					
- Butt kicks, reps	10 m	2					
- Hip rotation, reps	10 m	2					
- Side skips, reps	10 m	2					
- Backward run, reps	10 m	2					
- Shuttle runs, reps	5–5 m	2					
**Strength (10’ and 15’)**							
- Half-squat, reps		2 × 15	3 × 15	4 × 15	3 × 15	3 × 15	1 × 15
- Forward lunge, reps	10 m	2	3	4	3	3	1
- Nordic hamstring, reps		4	6	8	6	6	4
- Copenhagen exercise (right side), s		2 × 15 s	2 × 20 s	2 × 30 s	2 × 30 s	2 × 20 s	1 × 15 s
- Copenhagen exercise (left side), s		2 × 15 s	2 × 20 s	2 × 30 s	2 × 30 s	2 × 20 s	1 × 15 s
- Calve extension, reps		2 × 15	3 × 15	4 × 15	4 × 15	3 × 15	1 × 15
- Hip thrust, reps		2 × 15	3 × 15	4 × 15	4 × 15	3 × 15	1 × 15
**Plyometrics (7’ to 10’)**							
**-** Forward leap over a 25 cm obstacle, reps		-	1 × 6	2 × 6	3 × 6	2 × 6	2 × 6
- Side jump over a cone obstacle (right), reps		-	1 × 6	2 × 6	3 × 6	2 × 6	2 × 6
- Side jump over a cone obstacle (left), reps		-	1 × 6	2 × 6	3 × 6	2 × 6	2 × 6
- Forward leap over a 45 cm obstacle, reps		-	1 × 4	2 × 4	3 × 4	2 × 4	2 × 4
- Side jump over 35 cm obstacle (right), reps			1 × 4	2 × 4	3 × 4	2 × 4	2 × 4
- Side jump over 35 cm obstacle (left), reps			1 × 4	2 × 4	3 × 4	2 × 4	2 × 4
**Change-of-direction speed (7’ to 10’)**							
- shuttle runs (angle 180°)	10 m	1	2	3	4	2	1
- change-of-direction speed (angle 45°)	20 m	1	2	3	4	2	1
- change-of-direction speed (angle 90°)	20 m	1	2	3	4	2	1
**Core stability and balance (7’ to 15’)**							
- proprio bosu ball (right leg), s		2 × 15 s–15 s	3 × 20 s–10 s	3 × 30 s–10 s	2 × 45 s–15 s	2 × 45 s–15 s	1 × 45 s–15 s
- proprio bosu ball (left leg), s		2 × 15 s–15 s	3 × 20 s–10 s	3 × 30 s–10 s	2 × 45 s–15 s	2 × 45 s–15 s	1 × 45 s–15 s
- plank on swissball, s		2 × 15 s–15 s	3 × 20 s–10 s	3 × 30 s–10 s	2 × 45 s–15 s	2 × 45 s–15 s	1 × 45 s–15 s
- side plank with legs apart (Right side), s		2 × 15 s–15 s	3 × 20 s–10 s	3 × 30 s–10 s	2 × 45 s–15 s	2 × 45 s–15 s	1 × 45 s–15 s
- side plank with legs apart (Left side), s		2 × 15 s–15 s	3 × 20 s–10 s	3 × 30 s–10 s	2 × 45 s–15 s	2 × 45 s–15 s	1 × 45 s–15 s
- crunch on swissball, s		2 × 15 s–15 s	3 × 20 s–10 s	3 × 30 s–10 s	2 × 45 s–15 s	2 × 45 s–15 s	1 × 45 s–15 s
- lumbar extension, s		2 × 15 s–15 s	3 × 20 s–10 s	3 × 30 s–10 s	2 × 45 s–15 s	2 × 45 s–15 s	1 × 45 s–15 s

Reps, repetitions; s, seconds; m, meter;

### Estimation of the Menstrual Cycle Phases

As weekly measures of urinary luteinizing hormone and serum estrogen (E2: 17-estradiol) and progesterone (P4) concentrations were not possible in our study [33], the players’ menstrual cycle was determined using the calendar calculation method [34]. The players had to record the first day of menstruation on an Excel platform that was designed for the menstrual monitoring of players. The average cycle length of each player was determined from the three menstrual cycles preceding the study. We assumed that players had a regular ovulatory menstrual cycle if the standard deviation of the length of each cycle did not exceed 3 days [34]. We acknowledge this method did not allow us to determine if an athlete was experiencing a luteal phase deficiency and potentially anovulatory and not experiencing a natural menstrual cycle [35].

The players’ injuries were classified according to the methodological recommendations of Elliott-Sale et al., (2021) [33] into the early follicular phase (day 1 to 4), the late follicular phase (day 10 to 13) and the mid-luteal phase (day 20 to 23) based on an average cycle of 28 days [36].

### Injury Recording

Injuries were identified and classified in accordance with the FIFA Consensus Statement [37]. Injuries were recorded by the medical staff on a database containing type, location, severity, and phase of the menstrual cycle (early follicular phase, late follicular phase, mid-luteal phase and the other phases). Injuries were categorized into seven types according to the Consensus Statement [37]. These included fractures and stresses to joint (non-bone) bone and ligaments, muscles and tendons, contusions, lacerations and skin lesions, central/peripheral nervous system, and other injuries. We used the following twelve locations to identify affected areas: foot, ankle, lower leg, knee, thigh, hip/groin, upper limbs shoulder/clavicle, lumbar/sacrum/pelvis, head/face/neck/ cervix, abdomen and sternum/rib/dorsal. Finally, the severity of the injury was defined by the number of days absent: minimal injury (absence 1 to 3 days), slight (absence 4 to 7 days), moderate (absence 8 to 28 days), and serious (absence more than 28 days) [37].

### Statistical Analyses

The Shapiro–Wilk test was used to assess and confirm normality of data distribution. Data are presented as means and standard deviations (SDs). Schwartz’s Bayesian criterion was performed to classify players according to their performance during the pretest. Two-factor analysis of variance (ANOVA) (2 [group: NMT, ET] × 2 [time: pre, post]) was used to analyze the within and between group evaluation over the six-weeks of training. If a significant group-by time interaction effect occurred, Bonferroni adjusted post-hoc tests (t-tests) were computed. Cohen’s d (d) was calculated to quantify meaningful differences in the data with demarcations of trivial (< 0.2), small (0.2–0.59), medium (0.60–1.19), large (1.2–1.99), and very large (≥ 2.0) being used [38]. Percentage changes from pre-test to post-test were also calculated. Test re-test reliability of the variables was assessed using Cronbach’s model of ICCs and SEMs according to the method of Peltola [39].

Concerning injury data, the normality of the distributions of injuries between the ET and NMT groups for type of injury (muscle strains/contractures, ligament sprains/ruptures, contusions/haematoma/tissue bruising, fracture/dislocation, laceration, central/peripheral nervous system, tendinosis joint injuries), location of injury (upper body, lower body), mechanism of injury (contact, non-contact) and injury burden (minimal, minor, moderate, severe) was tested using Shapiro–Wilk tests. The p-values found, which were lower than 0.05 for almost all variables, indicated a non-normal distribution. It was therefore not possible to perform independent sample t-tests. Therefore, a non-parametric alternative, the Mann-Whitney U test, was used to investigate differences in the dependent variables for the two independent groups. All p-values were two tailed and the significance level was set at *p* < 0.05.

For the GPS data, we performed a t-test for independent samples to compare the measurements. Cohen’s d (d) was calculated to quantify meaningful differences in the data [38].

Data were analyzed using SPSS software (SPSS, version 22, Chicago; IL).

## Results

### Anthropometric Characteristics

The anthropometric characteristics of the study participants are displayed in Table 4. A significant main effect of time was observed for height (*p* = 0.004; d = 1.38) and body fat (*p* < 0.001; d = 1.96). However, the analysis indicated no significant group-by-time interactions (0.114 < *p* < 0.703; 0.17 < d < 0.70) for all anthropometric parameters.

**Table 4:** Effects of 6 weeks of neuromuscular (NMT) or endurance-dominated training (ET) on anthropometrics and body composition of study participants (mean ± SD)

Variables	Group	Pre	Post	Change %	Cohen’s d	ANOVA *p*-value (Cohen’s d)		
						Time	Group	Group × time interaction
Body mass (kg)	ET	60.2 ± 5.6	59.6 ± 5.7	-0.99	0.11	0.440(0.33)	0.314(0.44)	0.686(0.18)
	NMT	58.1 ± 4.5	57.9 ± 2.9	-0.34	0.05			
Height (cm)	ET	165.9 ± 5.3	166.3 ± 5.3	0.23	0.07	0.004(1.38)	0.478(0.31)	0.703(0.17)
	NMT	164.3 ± 5.2	164.8 ± 5.3	0.29	0.09			
Body fat (%)	ET	22.4 ± 3.0	20.4 ± 2.6	-8.84	0.71	< 0.001(1.96)	0.644(0.2)	0.114(0.7)
	NMT	21.3 ± 3.8	20.3 ± 3.4	-4.42	0.26			
BMI (kg/m²)	ET	21.9 ± 1.9	21.6 ± 1.6	-1.42	0.16	0.244(0.51)	0.684(0.18)	0.694(0.17)
	NMT	21.5 ± 1.9	21.4 ± 1.6	-0.70	0.08			

ET, endurance-dominated training group; NMT, Neuromuscular training group; BMI, body mass index

### Physical Fitness Tests

Data pertaining to physical fitness are displayed in Table 5. Significant main time effects (*p* < 0.001, 1.66 < d < 9.93) were observed for SJ, CMJ, 1-RM bench press and 1-RM squat, 5-m sprint, 10-m sprint, 30-m sprint, T-test, T-test with ball, RSSA, YYIRT 1 and LSPT.

**Table 5:** Effects of 6 weeks of neuromuscular (NMT) vs. endurance-dominated (ET) training programs on physical fitness (mean ± SD)

Variables	Group	Pre	Post	Change %	Cohen’s d	ANOVA *p*-value (Cohen’s d)		
						Time	Group	Group × time interaction
**5-m sprint** (s)	ET	1.19 ± 0.07	1.09 ± 0.07	-8.40	1.43	< 0.001(4.86)	0.002(1.49)	< 0.001(2.16)
	NMT	1.18 ± 0.08	0.92 ± 0.06	-22.03	3.68			
**10-m sprint** (s)	ET	2.08 ± 0.12	1.94 ± 0.09	-6.73	1.32	< 0.001(5.27)	0.007(1.26)	< 0.001(2.58)
	NMT	2.11 ± 0.13	1.69 ± 0.09	-19.91	3.78			
**30-m sprint** (s)	ET	5.07 ± 0.24	4.85 ± 0.19	-4.34	1.02	< 0.001(4.28)	0.052(0.88)	< 0.001(2.23)
	NMT	5.15 ± 0.28	4.44 ± 0.21	-13.79	2.87			
**T-test** (s)	ET	11.76 ± 1.03	10.86 ± 0.62	-7.65	1.06	< 0.001(2.82)	0.188(0.59)	0.024(1.03)
	NMT	11.94 ± 0.94	10.01 ± 0.44	-16.16	2.63			
**T-test with ball** (s)	ET	14.62 ± 1.43	13.49 ± 0.79	-7.73	0.98	< 0.001(2.33)	0.438(0.34)	0.084(0.77)
	NMT	14.88 ± 1.54	12.65 ± 0.58	-14.99	1.92			
**SJ** (cm)	ET	27.3 ± 3.3	29.6 ± 3.5	8.42	0.68	< 0.001(9.93)	0.159(0.62)	< 0.001(4.04)
	NMT	27.6 ± 3.1	33.1 ± 2.9	19.79	1.81			
**CMJ** (cm)	ET	31.1 ± 3.2	33.9 ± 3.3	9.28	0.89	< 0.001(7.78)	0.068(0.82)	< 0.001(2.21)
	NMT	32.9 ± 4.4	38.0 ± 4.3	15.66	3.28			
**YYIRT 1** (m)	ET	1396.7 ± 414.8	1583.3 ± 382.7	13.36	0.47	< 0.001(5.55)	0.546(0.29)	0.139(0.67)
	NMT	1466.7 ± 365.6	1703.3 ± 362.4	16.14	0.65			
**RSSA** (%)	ET	6.1 ± 2.5	4.6 ± 1.6	-23.97	0.68	0.001(1.66)	0.870(0.06)	0.287(0.46)
	NMT	6.7 ± 1.5	4.1 ± 1.7	-38.30	1.58			
**LSPT** (s)	ET	71.50 ± 6.54	59 ± 6.44	-17.48	1.93	< 0.001(5.78)	0.985(0)	0.019(1.08)
	NMT	74.42 ± 5.07	56.17 ± 6.04	-24.52	3.27			
**1-RM bench press** (kg)	ET	50.0 ± 7.5	55.0 ± 8.2	10	0.64	< 0.001(2.85)	0.738(0.142)	0.581(0.24)
	NMT	50.5 ± 6.6	56.2 ± 6.5	11,19	0.86			
**1-RM squat** (kg)	ET	110.0 ± 16.8	118.7 ± 14.8	7.88	0.55	< 0.001(4.98)	0.066(0.82)	< 0.001(2.53)
	NMT	113.3 ± 19.3	140.0 ± 12.6	23.53	1.63			

ET, endurance-dominated training group; NMT, neuromuscular training group; SJ, squat jump; CMJ, countermovement jump; YYIRT 1, yoyo intermittent recovery test level 1; RSSA, repeated-shuttle-sprint ability; LSPT, Loughborough soccer passing test total performance time; RM, repetition maximum

Significant group-by-time effects (0.001 < *p* < 0.019, 1.03 < d < 4.04) were observed for SJ, CMJ, 1-RM back squat, the 5-m sprint, 10-m sprint, 30-m sprint, T-test and LSPT.

The post-hoc tests revealed significant physical fitness increases in favor of the NMT group for SJ (*p* = 0.015, d = 1.08), CMJ (*p* = 0.015, d = 1.07), 1-RM back squat (*p* = 0.001, d = 1.55), 5-m sprint (*p* < 0.001, d = 2.61), 10-m sprint (*p* < 0.001, d = 2.77), 30-m sprint (*p* < 0.001, d = 2.04) and T-test (*p* = 0.001, d = 1.58).

### Training Load

Data of internal and external training loads are shown in Table 6. The internal load (RPE) was similar in the two experimental groups (1757 ± 255 AU in the ET group vs. 1736 ± 249 AU in the NMT group). The internal load of the two programs were not different (*p* = 0.067; d = 0.08). The external load of the two groups (expressed by GPS data) were not different in terms of the total distance, the distance at moderate pace (13–19 km/h), the distance at high intensity (19–23 km/h), and sprint distance (> 23 km/h). However, GPS data indicated significant differences in favor of the NMT group with regard to the number of sprints (*p* < 0.001; d = 2.87), acceleration (*p* = 0.006; d = 1.96) and deceleration (*p* = 0.009; d = 1.99).

**Table 6:** Indicators of external (GPS data) and internal load (RPE) of players determined during the 6 weeks preseason preparation period

	Indicators of external load (GPS data)								Indicators of internal load (session RPE)
	Group	TD	13–19 km	19–23 km	> 23 km	Sprint event	Acc event	Dec event	A.U.
**Week 1**	ET	4728 ± 2392	1905 ± 932	151 ± 97	28 ± 24	14 ± 7	15 ± 8	17 ± 8	1753 ± 207
	NMT	4577 ± 2194	2213 ± 1151	146 ± 93	35 ± 30	25 ± 15	20 ± 10	20 ± 10	1700 ± 197
**Week 2**	ET	5560 ± 2766	2622 ± 1329	140 ± 73	45 ± 37	11 ± 8	16 ± 9	15 ± 10	1862 ± 186
	NMT	5284 ± 2570	1986 ± 1074	174 ± 96	106 ± 57	23 ± 15	25 ± 15	25 ± 13	2030 ± 211
**Week 3**	ET	5448 ± 2672	1594 ± 912	135 ± 79	112 ± 61	16 ± 10	18 ± 9	18 ± 9	2076 ± 197
	NMT	5014 ± 2395	1823 ± 914	165 ± 93	112 ± 66	34 ± 20	27 ± 15	30 ± 18	2002 ± 191
**Week 4**	ET	5723 ± 2806	2142 ± 1088	137 ± 74	144 ± 88	17 ± 8	15 ± 8	17 ± 8	1712 ± 147
	NMT	5487 ± 2787	2158 ± 1068	147 ± 73	165 ± 100	30 ± 18	33 ± 19	28 ± 17	1640 ± 143
**Week 5**	ET	5787 ± 2978	2307 ± 1400	147 ± 75	133 ± 71	20 ± 9	19 ± 9	22 ± 11	1840 ± 197
	NMT	5442 ± 2817	1963 ± 1478	180 ± 92	168 ± 98	33 ± 25	35 ± 23	35 ± 24	1686 ± 165
**Week 6**	ET	5353 ± 3030	2545 ± 1364	195 ± 127	94 ± 59	22 ± 11	26 ± 15	27 ± 15	1304 ± 119
	NMT	5484 ± 3190	2281 ± 1569	216 ± 140	126 ± 73	34 ± 24	32 ± 20	31 ± 21	1360 ± 115
**Means**	ET	5433 ± 382	2186 ± 391	151 ± 23	93 ± 47	17 ± 4	18 ± 4	19 ± 4	1757 ± 255
	NMT	5215 ± 361	2071 ± 175	171 ± 26	119 ± 49	30 ± 5	28 ± 6	28 ± 5	1736 ± 249
p-value (Cohen’s d)		0.33(0.59)	0.525(0.38)	0.176(0.81)	0.370(0.54)	< 0.001(2.87)	0.006(1.96)	0.009(1.99)	0.067 (0.08)

ET, endurance-dominated training group; NMT, neuromuscular training group; RPE, ratings of perceived exertion; GPS, Global Positioning System; TD, Total Distance; Acc event, acceleration event; Dec event, deceleration event; AU, arbitrary unit; (mean ± SD).

### Injury Record

The injury data recorded during the different periods of the season are presented in Tables 7 and 8. In the documented season, 76 injuries were reported, including 53 injuries (70%) in the ET group and 23 injuries (30%) in the NMT group *(*Table 7*).* The total number of injuries recorded in the ET group was significantly higher compared to the NMT group throughout the season (*p* = 0.014). The majority of these injuries (62%) occurred during the follicular phase (10 injuries in early follicular phase and 37 injuries in late follicular phase) of the menstrual cycles of players in both groups (*p* = 0.021). Furthermore, the number of injuries recorded in the late follicular phase (*p* = 0.020) was significantly higher in the ET group (34%) compared to the NMT group (14%).

**Table 7:** Types of injuries

Injury type	Group	Injuries recorded	Injuries during the menstrual cycle			
			Early follicular phase	Late follicular phase	Mid-luteal phase	Other phases
**Muscle strains /contractures**	ET	12 (23%)	1 (14%)	8 (31%)	2 (13%)	1 (20%)
	NMT	3 (13%)	0 (0%)	3 (28%)	0 (0%)	0 (0%)
**Ligament sprains/ ruptures**	ET	14 (26%)	2 (29%)	10 (38%)	2 (13%)	0 (0%)
	NMT	3 (13%)	0 (0%)	2 (18%)	1 (17%)	0 (0%)
**Contusions/haematoma/** **tissue bruising**	ET	24 (45%)	4 (57%)	6 (23%)	10 (67%)	4 (80%)
	NMT	15 (65%)	2 (67%)	5 (45%)	5 (83%)	3 (100%)
**Fracture/ dislocation**	ET	0 (0%)	0 (0%)	0 (0%)	0 (0%)	0 (0%)
	NMT	1 (4%)	1 (33%)	0 (0%)	0 (0%)	0 (0%)
**Laceration**	ET	3 (6%)	0 (0%)	2 (8%)	1 (7%)	0 (0%)
	NMT	0 (0%)	0 (0%)	0 (0%)	0 (0%)	0 (0%)
**Central/peripheral nervous system**	ET	0 (0%)	0 (0%)	0 (0%)	0 (0%)	0 (0%)
	NMT	0 (0%)	0 (0%)	0 (0%)	0 (0%)	0 (0%)
**Tendinosis joint injuries**	ET	0 (0%)	0 (0%)	0 (0%)	0 (0%)	0 (0%)
	NMT	1 (4%)	0 (0%)	1 (9%)	0 (0%)	0 (0%)
Total injury ET		53 (70%)	7	26	15	5
Total injury NMT		23 (30%)	3	11	6	3
**Total injury**		76	10 (13%)	37 (49%)	21 (27%)	8 (11%)

ET, endurance-dominated training group; NMT, neuromuscular training group

**Table 8:** Injury data recorded during the different periods of the season

	Preseason period		Competitive period		Entire season	
	ET	NMT	ET	NMT	ET	NMT
**Injuries localizations**						
Upper body	1 (2%)	0 (0%)	2 (4%)	2 (9%)	3 (6%)	2 (9%)
Lower body	4 (7%)	2 (9%)	46 (87%)	19 (82%)	50 (94%)	21 (91%)
Total injuries	5 (9%)	2 (9%)	48 (91%)	21 (91%)	53	23
**Injury mechanisms**						
Contact	3 (6%)	2 (9%)	21 (40%)	16 (70%)	24 (45%)	18 (79%)
Non-contact	2 (4%)	0 (0%)	27 (50%)	5 (21%)	29 (55%)	5 (21%)
Total injuries	5 (10%)	2 (9%)	48 (90%)	21 (91%)	53	23
**Injury severity and number of absence days**						
***Minimal (1–3 days)***						
Number of injuries					29 (55%)	14 (61%)
Days lost					74	38
***Minor (4–7 days)***						
Number of injuries					15 (28%)	6 (26%)
Days lost					87	38
***Moderate (8–28 days)***						
Number of injuries					6 (11%)	2 (9%)
Days lost					84	29
***Sever (+ 29 days)***						
Number of injuries					3 (6%)	1 (4%)
Days lost					195	45
***Re-injury***					5	2
**Total days lost**					440	150
**Exposure time**(a)					4485	4485
**Burden** (b)					98.10	33.44
**Incidence injury rate**						
Number of injuries (c)	5	2	48	21	53	23
Exposure time training	585	585	3360	3360	3945	3945
Exposure time match	90	90	450	450	540	540
IIR (d)	7.40	2.96	12.59	5.51	11.82	5.13
Incidence injury rate **[**95% CI**]**	0.40 [0.08–2.06]		0.44 [0.26–0.73]		0.43 [0.26–0.70]	

ET, endurance-dominated training group; NMT, neuromuscular training group; (a) Exposure time calculated using number of female players per training session (around 12 players per group), Number of training session (around 205 for the examined period, including 48 training sessions during preseason period) and number of played matches (36 matches for the studied period, including 6 matches during preseason period); (b) burden = 1000 x (∑ days absent/ ∑ exposure hours) (Fuller CW. et al., 2006); (c) Number of injuries was recorded during training and matches. (d)IIR (Incidence injury rate) was calculated, IIR = 1000 x (∑ injuries/ ∑ exposure hours) (Fuller CW. et al., 2006). Season IIR (Incidence injury rate) including preseason and competition periods; CI, confidence interval

Regarding the type of injury, no significant differences were observed between the ET and NMT groups. The most frequent injuries were: contusion (24 injuries or 45%) in the ET group and 15 injuries (or 65%) in the NMT group (*p* = 0.242); ligament injuries (14 injuries or 26%) in the ET group and 3 injuries (or 13%) in the NMT group (*p* = 0.028); muscle injuries (12 injuries or 23%) in the ET group and 3 injuries (13%) in the NMT group (*p* = 0.178); lacerations (3 injuries or 6%) in the ET group; fracture and tendonitis (1 injury in the NMT group).

Most injuries in both groups were recorded in the lower limbs (93%) (*p* < 0.001) *(*Table 8*).* The injury mechanism was also recorded: in the ET group: 24 (or 45%) of injuries were contact related and 29 (or 55%) injuries were non-contact related; in the NMT group:18 (or 79%) of the injuries reported were contact related and 5 (or 21%) injuries were non-contact related *(*Table 8*).* Non-contact injuries were significantly higher (*p* < 0.001) in the ET group compared to the NMT group.

Regarding the time of absence (i.e., athlete availability) and the severity of injuries, there were 440 days of absence (burden = 98.10 days absent per 1000 h) in the ET group that included 29 minimal injuries (1–3 days), 15 minor injuries (4–7 days), 6 moderate injuries (8–28 days), 3 severe injuries (+ 29 days) and 5 recurrent injuries. In the NMT group, 150 days of absence (burden = 33.44 days absent per 1000 h) were recorded including 14 minimal injuries (1–3 days), 6 minor injuries (4–7 days), 2 moderate injuries (8–28 days), 1 severe injury (+ 29 days) and 2 recurrent injuries *(*Table 8*).* The injury burden among the ET group was significantly higher in comparison to that in the NMT group (*p* = 0.007).

The injury rate (per 1000 h of exposure) during the preseason was 7.40 in the ET group and 2.96 in the NMT group with an incidence rate ratio of 0.40 (95% CI, 0.08–2.06). However, the injury rate (per 1000 h of exposure) during the competition period was 12.59 in the ET group and 5.51 in the NMT group with an incidence rate ratio of 0.44 (95% CI, 0.26–0.73). The overall injury rate (per 1000 h of exposure) during the season was 11.82 and 5.13 in the ET and NMT group with an incidence rate ratio of 0.43 (95% CI, 0.26–0.70) ***(***Table 8***).*** During the preseason period, the incidence injury rate (IIR) did not show a significant difference between the ET and NMT groups (*p* = 0.187), however, the IIR was higher in the ET group compared to the NMT group during the competition period (*p* = 0.023). In addition, the overall IIR was higher in the ET group compared to the NMT group (*p* = 0.014).

## Discussion

To our knowledge, this is the first study that compared the effects of NMT versus ET applied during the preseason on measures of physical fitness and injury occurrence in highly trained [21] female soccer players. The main findings of this study were that six weeks of NMT showed larger improvements compared with ET on measures of physical fitness, and significantly fewer injuries were observed in the NMT compared with the ET group during the competitive season.

### Anthropometric Characteristics

Body composition and anthropometric parameters were not different in the ET versus NMT after the six weeks training program. Our findings are in agreement with previous studies which did not observe differences in body composition following six-weeks of NMT [40, 41].

### Physical Fitness Tests

The effects of NMT on performance measures has not been studied as frequently as NMT effects on injury prevention [6]. The few studies that exist demonstrated improvements in muscle strength, muscle power, linear and CoD speed in young athletes [42, 43]. Our study indicates that NMT showed significantly larger performance improvements than ET after the six weeks training program.

### Muscle Power and Muscle Strength

#### Vertical Jumps: SJ and CMJ

NMT is a multimodal exercise regime that includes plyometric exercises. Our findings demonstrate increases in SJ and CMJ performances in the NMT group with no improvements in the ET group, as also reported by Hammami et al. [6]. This improvement (SJ: +19%; CMJ: +15%) can most likely be explained by neural adaptive processes (e.g., improved motor unit recruitment, better synergistic muscle activation) [6]. Moreover, in this study, NMT was designed to include different types of jump exercises such as unilateral/bilateral and vertical/horizontal jumps. Research has shown larger performance improvements with a more variable plyometric exercise regime in young soccer players [44]. Indeed, jump training is characterized by utilizing plyometric activities conducted in the stretch-shortening cycle which involve the coupling of eccentric and concentric muscle actions [45] through jumping exercises and landings [46]. Jump training aims to improve the elastic component of the muscle-tendon unit [47], which has a direct influence on improving performance in high-intensity actions such as jumping and sprinting [48].

#### Maximal Strength: 1-RM

Our results indicate increased maximal strength (i.e., 1-RM) of the lower limbs (i.e., back-squat) in the NMT group with no improvements in the ET group, confirming the adaptations of contractile elements in the muscle groups involved and potentially translating into better-shot quality of the players [49]. However, our study did not show increases in 1-RM bench press, which can be explained by the specificity of the proposed NMT program that focused primarily on the lower body.

Our results are in agreement with previous studies that reported improvements in SJ performance [50, 51]. For example, Xiong et al. [49] showed that female table tennis players increased their 1-RM by 11.6% after 8 weeks of NMT. NMT is a multimodal exercise program that also contains exercises to improve maximal strength and power. These exercises will likely lead to improved lower extremity muscle strength and power, which is partly reflected in increased 1-RM performance post-training [49]. Maximal strength improvements can be caused by enhanced motor unit recruitment and/or firing frequency, leading to increased maximal voluntary strength. Given that we did not include electromyographic testing, previous assumptions on the underlying physiological mechanisms are speculative.

#### Linear Sprint and Change-of-direction Speed

Participants exposed to pre-season NMT experienced improvements in linear speed performance (5,10 and 30-m), CoD speed (T-test), and decreases in performance times in 5, 10 and 30-m sprints (-22%, -19% and − 13%) and T-test (without ball) (-16%). Our results are in agreement with previous studies on the effects of NMT on physical fitness demonstrating improvements in speed performance and CoD performance in young football players after a NMT program of six to eight weeks [42, 43]. The improvements in linear sprint speed in our study can be explained by better neuromuscular activation, reduced ground contact time and by better functional stiffness of the musculotendinous tissue [42]. The improvement in CoD speed can be explained by a better efficiency of the stretch-shortening cycle, i.e., a rapid passage in muscle action from the eccentric phase to the concentric phase [43]. In fact, Little and Williams [52] reported that accelerations, decelerations, CoDs, and maximum speed are specific neuromuscular qualities in professional soccer players, share common physiological and biomechanical determinants, and that improvements of these qualities are linked to the muscles recruited, the strength developed, the complex motor control and the coordination of several muscle groups.

Improvements in muscle strength and power contribute to the improvement in CoD speed performance, increases in eccentric muscle strength also improves CoD speed performance, mainly during the deceleration phases [6, 43]. This improvement in the CoD speed is believed to mainly be due to regulation by the nervous system [6]. In addition, Emonds and colleagues [19], demonstrated that reactive strength improves the speed and ability to change directions in female soccer players. Likewise, it has been shown that NMT impacts on muscle power and high intensity activities such as accelerations, decelerations, CoDs, jumps and sprints [43]. Our findings indicate improvements in scores for the T-Test with the ball for soccer players in both groups, although greater effects were measured in the NMT group.

### Soccer-specific Performance

#### RSSA and YYIRTL1

Significant main effects of time were observed for RSSA (d = 1.66) and YYIRTL1 (d = 5.55). Indeed, the two experimental groups (ET and NMT) improved their pre-post test performances for the RSSA test (-23%, -1.45; -38%, -2.57) and YYIRT 1 (+ 13%, + 186.66%; +16%, + 236.66%). However, our results show no significant group-by-time effect. The results of our study are in agreement with the result of the meta-analysis reported by [53], indicating that NMT greatly improves the ability to change directions and lower limb power, but with no effect on the ability to repeat sprints. Our results may be related to several benefits of NMT, such as movement control, muscle strength, coordination, agility, power, and dynamic stability, but without directly improving aerobic endurance. Some studies report no changes in aerobic capacity following a NMT program [54], while others report improvements in aerobic capacity [55]. These discrepancies may be associated with differences in the content and duration of the training program. The potential effect of NMT on improving anaerobic performance may be limited by exercises related to endurance and fatigue resistance which need to be verified in future research depending on the specificity of the repeated sprint test (sprint distance, number of sets, number of repetitions, recovery period) utilized.

#### The Loughborough Soccer Passing Test (LSPT)

Our study indicates improvements in LSPT performance in the NMT group (d = 1.08), which could be due to the effects of NMT on the improvement of several elements essential to performance in soccer skills, such as better coordination of the muscles involved in the passing movement, greater efficiency during the rotation movement, improved precision and timing during the execution of a movement of passes, enhanced cognitive-motor connections allowing a fast decision-making and better balance and the stability [20, 43]. Our results are in agreement with the findings of Zago et al. [56] of improved neuromuscular capacities related the technical performance of players [56]. A meta-analysis by Faude et al. [20] concluded that a NMT program improved the performance of specific soccer skills in young soccer players, likely by enhancing muscles to allow for better and more precise control of the soccer ball.

### Injuries

Soccer is a contact sport requiring repeated high-intensity efforts, which increase the predisposition of players to injuries, with or without contact [1]. Young players are not spared from this risk—but are exposed to a greater risk of injuries due to factors which are either extrinsic (e.g., training load, playing surface, rules) or intrinsic (e.g., hormonal changes rapid, change in body composition and size, change in neuromuscular function, flexibility, muscle imbalance, previous injuries) [1]. Research on injury to female soccer players (young and senior) is sparser than in male soccer players, and is mainly focused on anterior cruciate ligament (ACL) injuries due to their frequency in female soccer players [1].

#### Location of Injuries

Our study indicates that 94% and 91% of injuries were localized to the lower limbs in the ET and NMT groups, respectively, and are in agreement with other reports [6, 57]. That is, lower limbs are the most frequent regions for injuries in female and male players, youth and adult [6, 57]. This high prevalence of lower limb injuries most likely related to the nature of soccer activity; i.e., mainly involves the lower limb in the different actions performed by the player such as running, jumping, accelerating, decelerating, and changing direction [58]. In addition, longitudinal analysis of the change in hip strength and range of motion in young female soccer players demonstrated an increase in internal rotation and hip abduction with a decrease in hip external rotation and adduction which can impair neuromuscular control and compromise muscle activation during dynamic activities [59]. This can lead to an increased risk of lower limb injuries [59].

#### Type of Injuries

Our study identified contusions (contact and non-contact) as the most common injuries in both groups, with ∼ 45% in the ET group and ∼ 65% in the NMT group. It is difficult to assess completely the overall effect of NMT, which includes multiple components (e.g., strength, balance, agility), on the risk and type of injury. However, previous meta-analyses support the utility of NMT in reducing the risk of lower extremity and knee injuries, as well as a protective effect in reducing the risk of hip, thigh, knee and ankle injuries [60]. In fact, previous studies reported that contusions and contact injuries are highest in young female soccer players and vary between 47% and 94% [60]. To specify, ankle and knee sprains were the most common non-contact injuries in the ET group with approximately 26% of all injuries recorded, including 3 ACL injuries and 11 ankle sprains during the competition period. However, we did not record any ACL injuries in the NMT group, but ankle sprains accounted for 13% of all injuries occurring during the competition period. The absence of non-contact ACL injuries in the experimental group may be the effect of NMT which induces an increase in electromyographic activity in the hamstring muscles, in particular the semitendinosus. An early activation of the semitendinosus contributes to the reduction of excessive dynamic valgus and plays a protective role in the knee joint complex [61].

We recorded non-contact injuries related to muscular origin in ∼ 23% of participants in the ET group including 4 contractures, 6 elongations, and 2 muscle tears. A smaller number of participants in the NMT group (13%) experienced muscle injuries including 3 elongations. Other injuries accounted for less than 6% of all injuries, with 3 lacerations in the ET group, 1 tendinitis and 1 fracture in the NMT group. These results are consistent with those who regularly practice NMT integrated with stretching exercises and a reported reduction in the risk of sprains and strains [57].

Also, our results are in accordance with other reports showing that younger players seem to be more exposed to ligament sprains than to muscle injuries [57].

#### Mechanisms of Injuries

Neuromuscular risk factors contribute to the greater risk of non-contact injuries in female soccer players compared to male players [1, 62]. One reason for this due to anatomical features, where women “tend to land” with greater valgus of the knee, exposing them to a higher risk of rupture of the ACL [63]. The agonist/antagonist muscle imbalance of the hamstrings and quadriceps can also be a cause of ligament injury at the knee, because a high contraction of the quadriceps without the ability to control force by the hamstrings is sufficient to injure the ACL [62]. The low stability of the trunk and the lack of proprioceptive capacity is another factor that exposes female soccer players to injury of the ACL. Finally, the strong asymmetry of the moment and the valgus angle of the knee makes the player vulnerable to the risk of injury to the ACL [1]. Our findings on participants in the NMT group can be explained by the impact of the strength-dominated training program, as this type of training reduces the number of injuries including knee valgus in female soccer players [62].

#### Severity of Injuries

The severity of injuries was classified according to the number of days injured players were absent from regular team training or matches, as follows: minimal (absence 1 to 3 days), slight (absence 4 to 7 days), moderate (absence 8 to 28 days), and serious injury (absence more than 28 days) [37]. The data from our study shows that the NMT group recorded fewer absent days (33.44 days absent per 1000 h) compared to the ET group (98.10 days absent per 1000 h). The preventive effect of NMT may make it possible to reduce serious injuries and thus reduce the players’ number of absent days [61, 62, 64, 65]. These results confirm the data of several authors, who have demonstrated that NMT may lead to a reduction in the number of absent days of players from training and matches [64].

#### Incidence Rate of Injuries

The overall incidence of injuries throughout the season was 11.82 per 1000 h of exposure in the ET group and 5.13 per 1000 h of exposure in the NMT group. These injury rates include all training sessions and matches performed during the season (pre-season and competition period). The injury rates (per 1000 h of exposure) during the pre-season period were 7.40 in the ET group and 2.96 in the NMT group, and 12.59 for the ET group and 5.51 for the NMT group per 1000 h of exposure during the competition period.

In addition, our data also indicate incidence rate ratios of 0.40 (95% CI, 0.08–2.06) during the preseason period, 0.44 (95% CI, 0.26–0.73) during the competition period, and 0.43 (95% CI, 0.26–0.70) for the whole season. These findings indicate lower injury rates during different parts of the season in the NMT group compared to the ET group. Our data are in agreement with other studies on the effect of NMT in reducing injuries in young female soccer players [64, 65]. A meta-analysis by Sugimoto et al. evaluating exercise modes including strengthening, trunk, and balance exercises demonstrated greater ACL injury reduction in young female soccer players. NMT reduced injuries while also optimizing the physical fitness of female players [65].

#### Menstrual Cycle and Injuries

The impact of menstrual cycle phases on injury occurrence has been hypothesized to be related to the effects of changes in progesterone and estrogen hormone levels on ligament laxity and neuromuscular control of female players [33, 57, 66]. Our study recorded 47 injuries, with 62% of all injuries occurring during the follicular phase (21% in the early follicular phase; 79% in the late follicular phase) of the menstrual cycle of female players, including 12 injuries of muscular origin (9 in the ET group and 3 in the NMT group), 14 ligament injuries (12 in the ET group and 2 in the NMT group), 17 contusions (10 in the ET group and 7 in the NMT group), 2 lacerations (in the ET group), 1 fracture (in the NMT group), and 1 tendonitis (in the NMT group). This may, among other reasons, contribute to the occurrence of some non-contact injuries. It has been reported that ACL injuries occur more frequently during the late follicular phase with a high estrogenic hormonal concentration which is associated with significant ligament laxity [1, 67].

## Limitations

This research has certain limitations. The present study involved twenty-four female soccer players, which represents a small sample. Similarly, the expertise level of the players and their skill level (elite national or international players) could influence the study outcomes. Additionally, the lack of actual hormone measurements to determine the players’ phase within the menstrual cycle. Future studies should therefore take these limitations into account and specify the effects of NMT or ET on female soccer players’ physical fitness according to their expertise level and playing position.

## Conclusions

This comprehensive study demonstrates that a six-week NMT program during the preseason improves physical fitness and reduces the occurrence of injuries over the season in elite female soccer players compared to an ET training program. It is possible that the strength-dominated training during the preseason period improves the performance of players and reduces the risk of injury. Our findings may help soccer coaches to better choose preparation methods for female players during the preseason.

## Practical Applications

This study has important implications for coaches, particularly concerning the effective development of physical fitness and injury prevention. Indeed, our results indicate that a six-week multimodal NMT program with three weekly sessions including exercises to improve muscle strength and power, linear sprint and CoD speed, balance during the preseason resulted in significant improvements in physical fitness and injury prevention in highly trained female soccer players.

## Data Availability

All data supporting the findings of this study are available upon a reasonable request to corresponding authors.

## Abbreviations

ANOVA: Analysis of variance
CMJ: Countermovement
CoD: Change of direction
ET: Endurance-dominated training
GPS: Global positioning system
ICC: Intra-class correlation coefficients
CI: Confidence interval
LSPT: Loughborough soccer passing test
NMT: Neuromuscular training
RM: Maximal repetition
RSSA: Repeated shuttle sprint ability
session-RPE: Session rating of perceived exertion
SJ: Squat jump
YYIRT 1: Yo-Yo intermittent recovery test 1
